# compomics-utilities: an open-source Java library for computational proteomics

**DOI:** 10.1186/1471-2105-12-70

**Published:** 2011-03-08

**Authors:** Harald Barsnes, Marc Vaudel, Niklaas Colaert, Kenny Helsens, Albert Sickmann, Frode S Berven, Lennart Martens

**Affiliations:** 1 Proteomics Unit, Department of Biomedicine, University of Bergen, Norway; 2 Computational Biology Unit, UniComputing, Bergen, Norway; 3 Leibniz - Institut für Analytische Wissenschaften - ISAS - e.V., Dortmund, Germany; 4 Department of Medical Protein Research, VIB, B-9000 Ghent, Belgium; 5 Department of Biochemistry, Ghent University, B-9000 Ghent, Belgium

## Abstract

**Background:**

The growing interest in the field of proteomics has increased the demand for software tools and applications that process and analyze the resulting data. And even though the purpose of these tools can vary significantly, they usually share a basic set of features, including the handling of protein and peptide sequences, the visualization of (and interaction with) spectra and chromatograms, and the parsing of results from various proteomics search engines. Developers typically spend considerable time and effort implementing these support structures, which detracts from working on the novel aspects of their tool.

**Results:**

In order to simplify the development of proteomics tools, we have implemented an open-source support library for computational proteomics, called compomics-utilities. The library contains a broad set of features required for reading, parsing, and analyzing proteomics data. compomics-utilities is already used by a long list of existing software, ensuring library stability and continued support and development.

**Conclusions:**

As a user-friendly, well-documented and open-source library, compomics-utilities greatly simplifies the implementation of the basic features needed in most proteomics tools. Implemented in 100% Java, compomics-utilities is fully portable across platforms and architectures. Our library thus allows the developers to focus on the novel aspects of their tools, rather than on the basic functions, which can contribute substantially to faster development, and better tools for proteomics.

## Background

Proteomics has become an increasingly interesting field in the last decade, allowing the identification, quantification and characterization of proteins and peptides [1]. With this increased interest comes an increasing demand for high-performance bioinformatics solutions that help address the various data processing and data interpretation challenges in the field. And while these tools can vary substantially in purpose, a basic set of features are common to most of them. These include the handling of (FASTA) sequence databases and the heterogeneous headers they employ, protein and peptide sequences, the visualization of (and user interaction with) mass spectra and chromatograms, the parsing of various proteomics search engine results, and the storage of all relevant information in relational database systems. Developers typically have to spend considerable time and effort on implementing these basic support structures, shifting their focus away from the novel aspects of their tool. In order to provide such basic functionality in ready-made form, several groups have developed and released code libraries that can be reused. In the field of proteomics, ProteomeCommons released a Java-based I/O framework [2] for reading and converting certain file formats, C++ users can make use of the OpenMS [3] or Proteowizard [4] libraries, while Perl users can make use of InSilicoSpectro [5].

Despite the availability of the above packages however, no reasonably comprehensive, fully cross-platform library exists for the widely popular Java platform. Indeed, the ProteomeCommons I/O framework is the only Java library, and that contains only file access code, and none of the basic processing or end-user oriented visualization that takes up a considerable amount of time in developing user-friendly software. We have therefore been working steadily for several years on a general-purpose, highly reliably Java library for our own wide-ranging collection of user-oriented tools, including ms_lims [6] and DBToolkit [7] for storing and interacting with protein identification data; icelogo [8] for visualizing protein consensus sequences; Peptizer [9] for automating manual validation of MS/MS search results; Rover [10] for visualizing and validating quantitative proteomics data; MascotDatfile [11], OMSSA Parser [12] and XTandem Parser [13] for parsing results from Mascot [14], OMSSA [15] and X!Tandem [16] result files, respectively; jmzML [17] for parsing and visualizing mzML files [18]; and Fragmentation Analyzer [19] for analyzing MS/MS fragmentation data. By consistently building on this key library, and reusing it throughout all our distributed development, we have ensured a uniform way of presenting key user-interface elements, while substantially cutting back on development time through the reuse of shared functionality. Furthermore, this broad reliance on a single core library implicitly allows any improvement or addition to the compomics-utilities library to be directly available to all the tools using the library.

Given that the compomics-utilities library is already used in numerous software projects, it provides a broad range of functionalities and has been extensively tested and documented over the past several years. The result is a highly robust and simple to use codebase, aimed at component-based development rather than simply providing an amalgamate of methods. The library is here made available to the bioinformatics community as a whole, allowing anyone interested to benefit from its various production-grade features.

## Implementation

Compomics-utilities has been developed in 100% pure Java, is completely platform independent and only requires Java 1.5 (or newer) to work. In addition to the library itself, several demonstration applications showing how the library can be used have also been implemented. The platform independent Java binaries, additional documentation, the full source code of both demos and the library itself, along with real-life example code snippets are freely available at http://compomics-utilities.googlecode.com compomics-utilities is released under the permissive Apache2 open source license (http://www.apache.org/licenses/LICENSE-2.0.html) allowing for broad reuse of the code in other settings, including commercial ones.

## Results and Discussion

The compomics-utilities library contains a large amount of functionality, which is summarized in [Additional file 1]. Because it is impossible to describe in detail all functionality offered by compomics-utilities, we highlight three very different yet broadly applicable aspects of the library in the next sections: (i) interactive spectrum and chromatogram visualization; (ii) automatic generation of data access code; and (iii) the abstract modelling of typical proteomics (meta-)data and results. Together these examples illustrate how the compomics-utilities library can be used to support user interaction, back-end database development, and heterogeneous proteomics data handling. More comprehensive and detailed information on the various functionalities of compomics-utilities, along with short code examples and full source code, is available on the project web page (http://compomics-utilities.googlecode.com), where a cross-platform binary (including demos) can also be downloaded.

### Spectrum and chromatogram visualization

**Figure 1:**
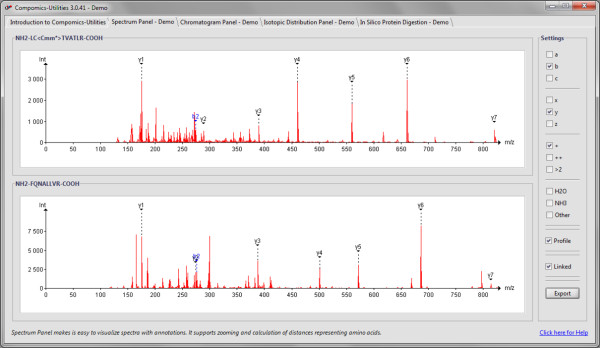
**Spectrum visualization in compomics-utilities**. The display supports both peak picking and zooming. If the peaks are annotated, the annotation can be switched on or off by the user. If more than one spectrum is shown on screen, the axes can be linked to support transitive zooming and rescaling. Finally, the spectra can be exported as images in common file formats.

Mass spectra (MS) and chromatograms are essential parts of proteomics data. In both cases the data consists of (x, y) coordinates, and visualizing these coordinates in graphical form helps understand the properties of the underlying data. The interactive display of spectra and chromatograms is therefore an essential element in most proteomics software tools, for which the compomics-utilities library contains ready-made components. The interactive display of spectra is handled through the SpectrumPanel component, which is created and fully initialized simply by calling its constructor. As a subclass of JPanel, any SpectrumPanel can be directly added to a Java frame or dialog without further intervention or configuration. An example containing two SpectrumPanels is given in Figure 1. Chromatograms are created in the same way, but now using the ChromatogramPanel constructor. In both cases it is possible to add optional annotations (such as fragment ion designations, or peptide elution times) to specific (x, y) coordinates as well. Both the spectrum as well as the chromatogram widget support a wide array of user interactions using mouse and keyboard, and allow export of their contents in several common image formats (e.g., for inclusion in manuscripts). Furthermore, these panels can also be linked easily, which means that if multiple panels are displayed simultaneously, zooming on one panel automatically also zooms all linked panels to the same location.

### Automatic generation of data access code

Since relational database access (for storage, update and retrieval) is a vital part of any type of user-oriented, high data volume application, compomics-utilities also comes with a highly robust code generator that automatically creates table-specific data access components in fully documented Java code. These generated components provide programmatic access to a single table in a relational database, wrapping the INSERT, UPDATE, SELECT and DELETE statements through the implementation of four key interfaces: Persistable, Updateable, Retrievable, and Deleteable. These four methods thus define the core behavior of the generated class and completely omit the use of SQL in the user's own code. The use of these four interfaces furthermore abstracts the actual nature of the data element or table being persisted, updated, accessed or deleted, allowing highly complex hierarchical structures to be stored in just a few (recursive) statements. This cuts back quite substantially on development time, while simultaneously providing a unified and highly robust way of handling data access from an application. This latter point is particularly relevant, since data access is a key part of such an application, and any bugs or errors introduced in this code will have wide-ranging repercussions. For this reason, the ms_lims data management system relies entirely on such generated components for all its communications with the back-end database. In order to generate code for an existing database table, it is sufficient to specify the database access credentials, the table name, and the desired output package for the generated class. The generator auto-detects all relevant table characteristics, including column types, (autogenerated) primary keys, and special auditing columns such as 'creationdate' and 'modificationdate'. The generated classes are transaction aware, and also handle peculiar column types like BLOB and CLOB transparently.

### Abstract modelling of proteomics (meta-)data

**Figure 2:**
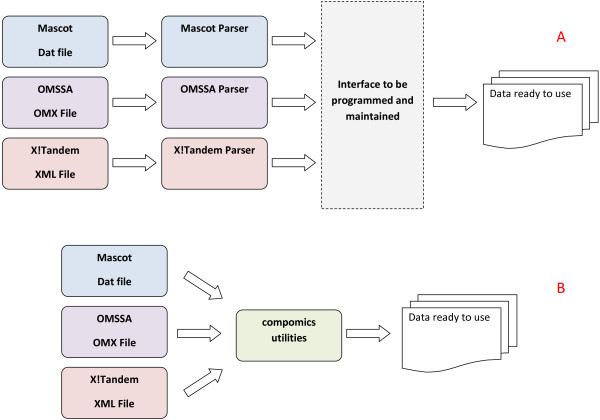
**Using compomics-utilities simplifies proteomics pipelines**. A standard pipeline (A) has to interact with multiple search engine output file formats and data structures before all data can be analyzed. With the use of compomics-utilities (B) the data from any of the specified input formats can all be loaded transparently and are ready for processing immediately.

Proteomics data is highly heterogeneous, spanning mass spectra, identified peptides and proteins, their quantification, and any and all associated experimental metadata such as sample and experiment information. On top of this complexity in basic data types, each type is often provided in one of multiple representations. Typical examples include FASTA database headers, mass spectrometry search engine output formats, and reporter-based quantification methods. These issues are addressed in compomics-utilities through abstract modelling of each data type, and this in turn is implemented transparently through auto-detection of supported formats for that data type. As a result, a FASTA header will be automatically parsed into its constituent parts without requiring any user or developer interaction, and the information from different search engine output formats can likewise be accessed through an abstracted interface directly without further effort (illustrated in Figure 2).

This framework thus makes it possible to have abstract access to information from the major parts of a proteomics workflow, saving development time, ensuring compatibility between tools, and reducing the complexity of (typically error-prone) data processing.

## Conclusions

By providing the basic building blocks of any proteomics informatics tool as a production-grade and permissively licensed open-source 100% pure Java API, the compomics-utilities library greatly simplifies the development of Java-based bioinformatics applications within the field of proteomics. Because compomics-utilities has been developed over several years, and is used in a large variety of production applications, it is thoroughly tested and reliable. Additionally, all code is well-documented and open-source, and is specifically engineered to be straightforward to incorporate into other projects. Finally, the fact that the library is already in active use in such a broad range of published software, helps ensure that the library will be both maintained as well as extended and expanded over time.

## Availability and requirements

**Project name**: compomics-utilities

**Project home page**: http://compomics-utilities.googlecode.com

**Operating system(s)**: Platform independent

**Programming language**: Java

**Other requirements**: Java 1.5 or newer

**License**: Apache License 2.0 (http://www.apache.org/licenses/LICENSE-2.0.html)

**Any restrictions to use by non-academics**: none

## List of abbreviations

OMSSA: the Open Mass Spectrometry Search Algorithm; MS: Mass Spectrometry; MS/MS: Tandem Mass Spectrometry; SQL: Structured Query Language

## Authors' contributions

All authors except FSB and AS participated in the implementation of compomics-utilities. All authors contributed in the writing of the manuscript. All authors read and approved the final manuscript.

## Supplementary Material

Additional File 1 **Compomics-utilities features**. A list of compomics-utilities features Click here for file
