# Supplementary material for: compomics-utilities: an open-source Java library for computational proteomics
Source: BMC Bioinformatics. 2011 Mar 8;12:70. doi: 10.1186/1471-2105-12-70 (PMC3060842; doi:10.1186/1471-2105-12-70)
Supplement: Additional File 1 — Compomics-utilities features. A list of compomics-utilities features [file 1471-2105-12-70-S1.PDF]

Additional File 1: List of compomics-utilities features:

<u>Proteomics:</u>

- GraphicsPanel
    - for:
        - spectra (both profile and centroid)
        - chromatograms
        - (multiple) isotope distributions
    - with :
        - peak annotation with annotation filtering
        - support for multiple datasets in the same plot
        - peak selection/peak picking with distance calculation between peaks, automatically matching amino acid residue masses (with and without PTMs) for spectra
        - drag-and-drop zooming
        - mouse-over tooltips for each peak
        - linking multiple GraphicsPanel for zooming (when one panel is zoomed, all linked panels will zoom to exactly the same range)
        - user defined color selection
        - export to common image file formats, svg, png, pdf, etc.

- Isotope distribution calculation and visualization
    - Simultaneously display multiple datasets/peptide sequences (e.g. isotopic labeled peptides)
    - with different charges
    - with different number of neutrons added (e.g. due to stable isotope labeling)

- Mass calculation
    - based on a sequence in IUPAC format, or element composition
    - handles monoisotopic biochemical element masses and monoisotopic single-letter amino acid masses, but also supports user-defined alphabets and masses
    - also implemented as a ready-to-deploy Servlet

- NucleotideSequence
    - read from/to FASTA format
    - read from sequence of nucleotides
    - includes mass calculation
    - translation into 6 reading frames with stop codons
    - calculation of the reverse complementary strand
    - translate the specified DNA sequence into a single String of amino acids, starting from a specified base
    - Configurable translation table

- Protein/peptide sequence

    - calculate GRAVY scores
    - estimate Meek HPLC retention score for AA residues
    - Fully supports modifications (both terminal and internal modifications, including multiple modifications per residue)
    - calculate mass and m/z
    - can be created from an annotated sequence, including modifications; e.g., Ace-MATHM<Mox>PIR-COOH
    - can be created from FASTA sequence
    - can be created from amino acid sequence
    - truncation of sequence:
        - N-terminal truncation
        - C-terminal truncation
        - both sides
    - calculate molecular formula
    - calculate isotope distribution

- Enzymatic digestion
    - simple enzymes:
        - residues after which cleavage will occur
        - residues which inhibit cleavage
        - number of allowed missed cleavages
        - N- or C-terminal cleavage
    - Bi-functional enzymes
        - enzyme with a dual specificity; the N-terminus of a resultant peptide will be derived from one cleavage pattern, while the C-terminus will be derived from another cleavage pattern, mimicking serial digest (e.g., *in vivo* followed by *in vitro* cleavage)
    - RegExEnzyme
        - digestion based on a regular expression pattern
    - Load Enzyme properties from Mascot formatted text files, or define on-the-fly

- Protein header parsing
    - Fully parses a wide variety of FASTA format header lines
    - Automatically detects and parses a broad range of headers, from UniProtKB/Swiss-Prot, UniProtKB/TrEMBL (multiple versions, including the latest HUPO PSI standard formatting), NCBI and Ensembl formatted FASTA headers to often-used variants for Drosophila and Arabidopsis species specific databases

<u>Standardized Customizable Objects for Proteomics Experiments:</u>

- Standardized customizable objects that can abstract the representation of:
    - Biological samples
    - Mass spectrometry results
    - Identification output (search engine independent)

    - Quantification process based on reporter ions (technology independent)
- Automated generation of Standardized customizable objects based on user designed xml files for:
    - Glycans
    - Enzymes
    - PTMs
    - Reporter ion based quantification methods
- Automated generation of Standardized customizable objects based on files for:
    - Mascot
    - OMSSA
    - X!Tandem
    - Mgf files

<u>General utilities of interest:</u>

- automatic code generation of database accessor based on existing tables
    - Fully automatic code generator that creates robust, ready-to-compile and fully documented Java classes to interact with a database table
    - All generated classes implement a set of interfaces that define their behaviour, allowing the high-level abstraction of database interaction, regardless of table or content
    - Requires only a database table and output package name for the generated class as input
    - Automatically detects all relevant table information: column types, (automatically generated) primary keys, and special columns such as 'creationdate' and 'modificationdate'. The latter are automatically used for logging insert and update operations, respectively
    - Direct access to automatically generated primary keys after insert operation
    - Generated code is transaction aware
    - Transparently handles BLOB and CLOB data types

- methods for exporting graphics components to common image file formats
    - svg, png, pdf, etc.

- generic interface for the parsing of command-line arguments, options and flags
    - Simple but powerful mechanisms for interpreting a user-specified command-line
    - Efficiently enables unified command-line structure and definition across applications
